# Alleviation of hippocampal necroptosis and neuroinflammation by NecroX-7 treatment after acute seizures

**DOI:** 10.3389/fphar.2023.1187819

**Published:** 2023-08-02

**Authors:** Yihyun Roh, Su Bin Lee, Minseo Kim, Mi-Hye Kim, Hee Jung Kim, Kyung-Ok Cho

**Affiliations:** ^1^ College of Medicine, The Catholic University of Korea, Seoul, Republic of Korea; ^2^ Department of Medical Laser, Graduate School, Dankook University, Cheonan, Republic of Korea; ^3^ Department of Physiology, College of Medicine, Center for Human Risk Assessment, Dankook University, Cheonan, Republic of Korea; ^4^ Department of Pharmacology, Catholic Neuroscience Institute, Institute for Aging and Metabolic Diseases, College of Medicine, The Catholic University of Korea, Seoul, Republic of Korea; ^5^ Department of Biomedicine and Health Sciences, The Catholic University of Korea, Seoul, Republic of Korea

**Keywords:** NecroX-7, neuroinflammation, neuroprotection, necroptosis, excitotoxicity, seizure, hippocampus

## Abstract

Temporal lobe epilepsy (TLE) is one of the most common neurological disorders, but still one-third of patients cannot be properly treated by current medication. Thus, we investigated the therapeutic effects of a novel small molecule, NecroX-7, in TLE using both a low [Mg^2+^]_o_-induced epileptiform activity model and a mouse model of pilocarpine-induced status epilepticus (SE). NecroX-7 post-treatment enhanced the viability of primary hippocampal neurons exposed to low [Mg^2+^]_o_ compared to controls in an MTT assay. Application of NecroX-7 after pilocarpine-induced SE also reduced the number of degenerating neurons labelled with Fluoro-Jade B. Immunocytochemistry and immunohistochemistry showed that NecroX-7 post-treatment significantly alleviated ionized calcium-binding adaptor molecule 1 (Iba1) intensity and immunoreactive area, while the attenuation of reactive astrocytosis by glial fibrillary acidic protein (GFAP) staining was observed in cultured hippocampal neurons. However, NecroX-7-mediated morphologic changes of astrocytes were seen in both *in vitro* and *in vivo* models of TLE. Finally, western blot analysis demonstrated that NecroX-7 post-treatment after acute seizures could decrease the expression of mixed lineage kinase domain-like pseudokinase (MLKL) and phosphorylated MLKL (p-MLKL), markers for necroptosis. Taken all together, NecroX-7 has potential as a novel medication for TLE with its neuroprotective, anti-inflammatory, and anti-necroptotic effects.

## Introduction

Epilepsy is a common neurological disorder affecting people of all ages (World Health Organization, 2022). Among various types of epilepsies, temporal lobe epilepsy (TLE) is often refractory to anti-epileptic drugs with no cure (Tellez-Zenteno and Hernandez-Ronquillo, 2012), requiring the development of novel drugs that can ameliorate key pathologies of TLE. Abrupt and excessive neuronal excitability in the brain is thought to contribute to hippocampal sclerosis, a characteristic pathological finding in TLE patients (Steve et al., 2014; Tai et al., 2018). In animal models recapitulating TLE, prolonged seizure activities called status epilepticus (SE) can trigger multifaceted processes in the hippocampus, resulting in neuronal deaths that feature both apoptosis and necrosis (Sloviter et al., 1996; Sankar et al., 1998; Fujikawa et al., 2000b). Necroptosis, an inflammation-associated novel mode of cell death, has been proposed as one of the complicated mechanisms for neuronal death after SE (Dingledine et al., 2014; Choi et al., 2021). Given that neuroinflammation, including reactive astrocytosis and microglial activation, is frequently observed in the hippocampus after SE (Vezzani et al., 2011; Cho et al., 2019), inflammation and neuronal death can have intricate relationships in neurological diseases such as epilepsy (Yu et al., 2021).

NecroX-7 (also known as MIT-001), an indole-derived small molecule that belongs to the NecroX compound family, primarily inhibits the generation of mitochondrial reactive oxygen species (ROS) (Kim et al., 2010). NecroX-7 has demonstrated therapeutic efficacies in a wide range of diseases including graft-versus-host disease (GVHD) (Im et al., 2015), chemotherapy-associated mucositis (Im et al., 2019), acetaminophen-induced liver injury (Park et al., 2013), and myocardial (Hwang et al., 2018), renal (Jin et al., 2016), and hepatic ischemia-reperfusion injuries (Choi et al., 2010; Lee et al., 2016). These significant protective effects shown in multiple cell types are mainly derived from its anti-necrotic and anti-inflammatory properties, which are important pathological findings also shown in TLE. However, there has been no report evaluating possible applications of NecroX-7 in central nervous system (CNS) diseases including TLE.

Here we investigated whether NecroX-7 could have an impact on SE-induced hippocampal neurodegeneration using *in vitro* low [Mg^2+^]_o_-induced neurotoxicity and *in vivo* pilocarpine-induced SE models of epilepsy. We found that post-treatment of NecroX-7 after seizures, but not pre-treatment, had neuroprotective effects in the hippocampus. NecroX-7 post-treatment could alleviate low [Mg^2+^]_o_-induced hippocampal neuronal synaptic losses in a dose-dependent manner. In addition, reactive gliosis was significantly downregulated by NecroX-7 treatment after acute seizures in both *in vivo* and *in vitro*. Finally, the hippocampal expression of mixed lineage kinase domain-like pseudokinase (MLKL) and phosphorylated MLKL (p-MLKL) was significantly attenuated by NecroX-7 post-treatment, demonstrating an anti-necroptotic effect of NecroX-7 after acute seizures.

## Materials and methods

### Animals

Male 6-week-old C57BL/6N mice or pregnant rats (KOATECH, Pyungtaek, Korea) were bred at a constant temperature of 22°C ± 1°C in a light-controlled room with food and water *ad libitum*. All animal trials were approved by the Ethics Committee of the Catholic University of Korea (CUMS-2020-0103-04, CUMS-2022-0078-02) and Dankook University (DKU-22-047) and were carried out following the National Institutes of Health Guide for the Care and Use of Laboratory Animals (NIH publication no. 80-23, revised 1996).

### Primary cell culture and low [Mg^2+^]_o_-induced epileptiform activity

Rat hippocampal neurons were cultured as previously described (Choi et al., 2021; Hong et al., 2022). At embryonic day 19, maternal rats were anesthetized with 16.5% urethane (0.8 mL/100 g) and fetal hippocampal tissue was isolated in Ca^2+^- and Mg^2+^-free HEPES-buffered Hanks' salt solution (HHSS). The hippocampus was dissociated by trituration through a flame-narrowed Pasteur pipette and placed in Neurobasal medium with 2% B27 supplement, 0.25% Glutamax Ⅰ and penicillin/streptomycin/amphotericin B (100 U/mL, 100 μg/mL, 0.025 μg/each, respectively) for pure neurons or added to DMEM medium containing 10% FBS, 10% horse serum, and penicillin/streptomycin/amphotericin B (100 U/mL, 100 μg/mL, 100 μg/mL, respectively) for neuron-glia co-cultures. Cells were seeded at 1.1×10^5^/well on 25 mm cover glasses coated with matrigel (0.2 mg/mL; BD Biosciences, San Jose, CA, USA) for immunocytochemistry or 0.5×10^5^/well on 96 well plates for MTT assay. Then, neurons were cultured at 37°C in 10% CO_2_ and 75% of the culture medium was replaced after 3 days and 7 days, respectively. On day 9 after the cell-seeding, cultured rat hippocampal neurons were exposed to 0.9 mM [Mg^2+^]_o_ medium (CON) or 0.1 mM [Mg^2+^]_o_ medium (Low Mg^2+^) for 24 h to induce high-frequency epileptiform discharges that mimics acute seizures (Kim et al., 2008).

### Pilocarpine-induced mouse model of SE and sample preparation

A mouse SE model was generated as previously described (Jeong et al., 2011; Cho et al., 2015; Kim and Cho, 2018). In short, scopolamine methyl nitrate (2 mg/kg; Sigma-Aldrich, St. Louis, MO, United States, S2250) and terbutaline hemisulfate salt (2 mg/kg; Sigma-Aldrich, T2528) were administered intraperitoneally (i.p.) 30 min prior to pilocarpine hydrochloride (280 mg/kg; i. p., Sigma-Aldrich, P6503) injection. After pilocarpine was injected, behavioral seizures were carefully observed according to the modified Racine scale (Racine, 1972) and the time when the mouse exhibited the first grade 3 seizures (Seizure onset) and the time when the mouse started to demonstrate continuous motor seizures (SE onset) were recorded to assess the seizure kinetics in the groups. Mice with continuous generalized convulsive seizures higher than grade 3 were identified as having shown SE and were subjected to further studies. Diazepam (10 mg/kg, i. p.) was injected 3 h after the onset of SE to cease behavioral seizures. Then, they were housed in an incubator (30°C) and fed with water-moistened chow to facilitate recovery. After 2 days, they were sent back to the original environment.

After the final NecroX-7 treatment, animals were anesthetized with ketamine (50 mg/mL) and xylazine (23.3 mg/mL) mixture (4:0.5), followed by transcardial perfusion with saline. After the brain was removed, hemibrains were postfixed in 4% paraformaldehyde (pH 7.4) for 3 days, then immersed in 30% sucrose solution for 3 days. Then, the brains were frozen in liquid nitrogen and serial hippocampal coronal sections (30 μm thick) were made with a cryostat (Leica, Wetzlar, Germany, CM 1850) for staining. For the other half of the brain, the hippocampus was frozen under liquid nitrogen for western blotting.

### Drug treatment

NecroX-7 was provided by MitoImmune Therapeutics Inc. Cells were allocated into 4 groups: vehicle, 10 μM, 30 μM, or 100 μM NecroX-7. In the pretreatment groups, cells were exposed to either vehicle or NecroX-7 for 1 h prior to 0.1 mM [Mg^2+^]_o_ treatment (Kim et al., 2008; Kim et al., 2016). In the post-treatment group, cells started to be exposed to either vehicle or NecroX-7 at 1 h after 0.1 mM [Mg^2+^]_o_ medium–induced epileptiform activity until the end of the experiment. MK-801 (10 μM), an NMDA receptor blocker known to have neuroprotective effects, was pre-treated 30 min before exposure to low [Mg^2+^]_o_ medium as a positive control.

Pilocarpine-injected mice were allocated into 3 groups: placebo, 20 mg/kg, or 40 mg/kg of NecroX-7. One hour after diazepam injection, each group was given intraperitoneal injection of placebo or drug solutions, which was prepared by dissolving the powder in 5% dextrose. Mice were daily administered the same dose of NecroX-7 or placebo for the following 7 days after pilocarpine injection. On the 7^th^ day, mice were sacrificed after the final injection of the drug.

### 3-[4,5-Dimethylthiazol-2-yl]-2, 5-dipheyltetrazolium bromide (MTT) assay

On day 9 after the cell-seeding in 96-well plates, the pretreatment group was treated with NecroX-7 (10 μM, 30 μM, and 100 μM) for 1 h and then cultured with Neurobasal medium containing L-glutamine, 2% B27 supplement, 0.25% Glutamax Ⅰ and penicillin/streptomycin/amphotericin B (100 U/mL, 100 μg/mL, 0.025 μg/mL, respectively) for 24 h. The post-treatment group was cultured in the same condition and then treated with NecroX-7 (10 μM, 30 μM, and 100 μM) 1 h later. After 24 h, neurons were treated with 20 μL of MTT (0.5 mg/mL) for 4 h. The absorbance at 570 nm was recorded using a microplate reader (Bio-Rad, Hercules, CA, United States).

### Histologic assessments

Immunocytochemistry was performed after culturing primary neurons for 9 days. After the fixation with pre-chilled methanol, cells were permeabilized with 0.3% Triton X-100 for 5 min. Then, cells were blocked with 10% bovine serum albumin (BSA) for 1.5 h at room temperature. After blocking, the primary antibody was incubated at 4°C overnight. Antibodies used were mouse anti-microtubule-associated protein 2 (MAP2) (1:200; Sigma-Aldrich), rabbit anti-postsynaptic density protein (PSD95) (1:200; Abcam, Cambridge, MA, United States), mouse anti-glial fibrillary acidic protein (GFAP) (1:200; MAB360, Millipore, Burlington, MA, United States) and rabbit anti-ionized calcium binding adaptor molecule 1 (Iba1) (1:1000; 019-1971, Wako, Richmond, VA, United States). The next day, Alexa Fluor 488-conjugated anti-rabbit IgG (1:500; Invitrogen, Carlsbad, CA, United States) and Alexa Fluor 555-conjugated anti-mouse IgG (1:500; Invitrogen) were incubated for 1.5 h at room temperature. After the counterstaining with DAPI, coverslips were mounted on slides using VECTASHIELD mounting medium (Vector Laboratories, Inc., Burlingame, CA, United States) and observed under confocal microscopy. For reactive gliosis after pilocarpine-induced SE, immunohistochemistry was performed using free-floating methods as described in previous studies (Brulet et al., 2017; Jang et al., 2019; Cho et al., 2020). Briefly, the same protocol with immunocytochemistry was followed except for the incubation with peroxidase-conjugated secondary antibodies and 0.05% 3,3′-diaminobenzidine (DAB) for color development. The reaction was terminated by 0.05 M trizma buffer and sections were mounted with dibutylphthalate polystyrene xylene (DPX), and observed under a light microscope (Olympus, Tokyo, Japan, BX51). For the evaluation of SE-induced neuronal death, Fluoro-Jade B (FJB) staining was performed (Choi et al., 2022). Slides with coronal hippocampal sections were placed in 0.06% KMnO4 solution for 7 min, followed by the incubation in 0.001% FJB (Biosensis, Thebarton, Australia, TR-150-FJB) for 30 min. Afterwards, slides were dehydrated with serial ethanol and xylene. Finally, sections were mounted with DPX and visualized using Lionheart FX system (Biotek, Winooski, VT, United States).

### Confocal imaging

Primary neurons were transferred to a confocal microscope (LSM 700; Carl Zeiss, Jena, Germany) and observed through a ×60 objective (numerical aperture, 0.8). A total of eight cross-sections were obtained at 1 μm intervals along the *z*-axis of the cell using the z-stack imaging technique and were combined to obtain one image. Using an argon ion laser, the GFP is excited at 488 nm and emits light at 519 nm (10 nm band pass). The RFP is excited at 555 nm (HeNe laser) and emitted at 565 nm.

### Microscope analysis and quantification

FJB-positive or immunoreactive cells were quantified as previously described (Choi et al., 2022). The number of FJB-positive cells was counted in every 12th hippocampal section, which was summed up and multiplied by 24 to estimate the total number of FJB-expressing cells in an animal. The CA1 and CA3 pyramidal cell layer (PCL) was demarcated by the point of rapid widening of PCL thickness and the larger soma size of CA3 pyramidal cells. The hilus was defined as the triangular area formed by connecting endpoints of the upper and lower granule cell layer (GCL) but excluding the CA4 PCL. As for quantitative analysis of reactive gliosis, the percentage of Iba1-and GFAP-immunoreactive areas in the dentate gyrus (DG) was analyzed using NIH ImageJ software. Sections with damaged regions in the hippocampal subfields were excluded from the analysis and samples containing two or more sections with damaged subfields were also excluded from the analysis. Briefly, background intensity was determined by measuring the pixel intensity of non-tissue area in each image. Then, Iba1-and GFAP-immunoreactivity were determined using the background intensity as a threshold for reactivity. Finally, the percentage of the marker-immunoreactive area over DG area value for all the images was calculated, and then those values were averaged for each mouse. The Iba1-immunoreactive area in CA1 and CA3 subfields of the hippocampus was evaluated using the same protocol as described above. For primary cultured neurons, the integrated intensity was quantified as the sum of pixel values of target immunoreactivity in an image of GFAP- or Iba1-positive areas using ImageJ software (NIH, United States) (Hong et al., 2021). Analysis of PSD95 puncta superimposed on MAP2 immunocytochemistry was done by quantitative measurements of the number of PSD95 puncta per 0.1 mm dendrite recognized by MAP2.

### Western blotting

Western blotting was performed as described in our previous work (Choi et al., 2021). Briefly, the hippocampus and cultured rat hippocampal neurons were lysed in ProEX™ CETi lysis buffer (TransLab Biosciences, Carpentersville, IL, United States) and ProPrep™ lysis buffer (iNtRON Biotechnology, Sungnam, Korea), respectively. Equal amounts of protein (40–60 µg for the hippocampus and 15–20 µg for cultured rat hippocampal neurons) were separated onto a 12% and 5% SDS-polyacrylamide gels, respectively, and were then transferred onto the polyvinylidene difluoride membranes. The membranes were incubated with 3% BSA for an hour at room temperature. Then the membranes were placed in anti-MLKL (1:1,000, Cell Signaling Technology, Danvers, MA, United States, 14993S), anti-p-MLKL (1:1,000, Cell Signaling Technology, Danvers, MA, United States, 18,640), or anti-phosphorylated RIP3 (1:500, Abcam, Cambridge, MA, United States, ab195117) at 4°C overnight. The next day, horseradish peroxidase-conjugated anti-rabbit IgG (1:2,000, GeneTex, Irvine, CA, United States) was added for 2 h. Afterwards, bands were detected by enhanced chemiluminescence detection kit (Elpis Biotech, Inc., Lexington, MA, United States). For loading controls, the membranes were stripped with Restore Western Blot Stripping buffer (Thermo Scientific, Waltham, MA, United States) and re-probed with mouse monoclonal anti-glyceraldehyde-3-phosphate dehydrogenase (GAPDH, 1:1,000, Santa Cruz Biotechnology, Inc., Dallas, TX, United States, SC-32233) for animal tissues and mouse monoclonal anti-β-actin (1:2,000; Santa Cruz Biotechnology, Inc., Dallas, TX, United States) for cultured rat hippocampal neurons. Then, the membranes were incubated with horseradish peroxidase-conjugated secondary antibodies (1:2000, GeneTex) and visualized under ImageQuant LAS 4000 (Fujifilm, Tokyo, Japan). Finally, relative quantification of immunoreactivity of each band was carried out using the method of densitometric analysis.

### Statistical analysis

Data are presented as mean ± standard error of the mean (SEM) and statistical significance was assessed using GraphPad Prism 9 software (GraphPad Software Inc.) or SPSS 12.0. Experimental groups were randomly assigned, and the exact sample size is presented in the figure legends. To reduce the experimental bias, analyses were performed by a researcher blind to the experimental groups. Body weight changes of pilocarpine-induced SE model mice were analyzed using Repeated measures one-way ANOVA, while seizure onset and SE onset after pilocarpine injection were analyzed using one-way ANOVA with Tukey’s multiple comparisons and Kruskal-Wallis test with Dunn’s multiple comparisons test, respectively. For FJB staining results, statistical differences were mainly determined by Kruskal–Wallis test with Dunn’s multiple comparisons test as normal distribution was not assumed. For DG analysis, Brown-Forsythe one-way ANOVA with unpaired *t*-test with Welch’s correction was performed as normal distribution was assumed but equal variance was not assumed. For analysis of Iba1 and GFAP immunohistochemistry, One-way ANOVA with Tukey’s multiple comparisons test was used when normal distribution was assumed. In case normal distribution was not assumed, Iba1 in CA3 subfield, Kruskal-Wallis test with Dunn’s multiple comparisons test was done. For western blot analysis, one-way ANOVA with Newman-Keuls multiple comparisons test was used. For *in vitro* results, one-way ANOVA with Bonferroni post-hoc test was performed. The level of statistical significance was set as 95% confidence interval *p* < 0.05 or 99% confidence interval *p* < 0.01.

## Results

### NecroX-7 treatment protects seizure-induced hippocampal neuronal death

**FIGURE 1:**
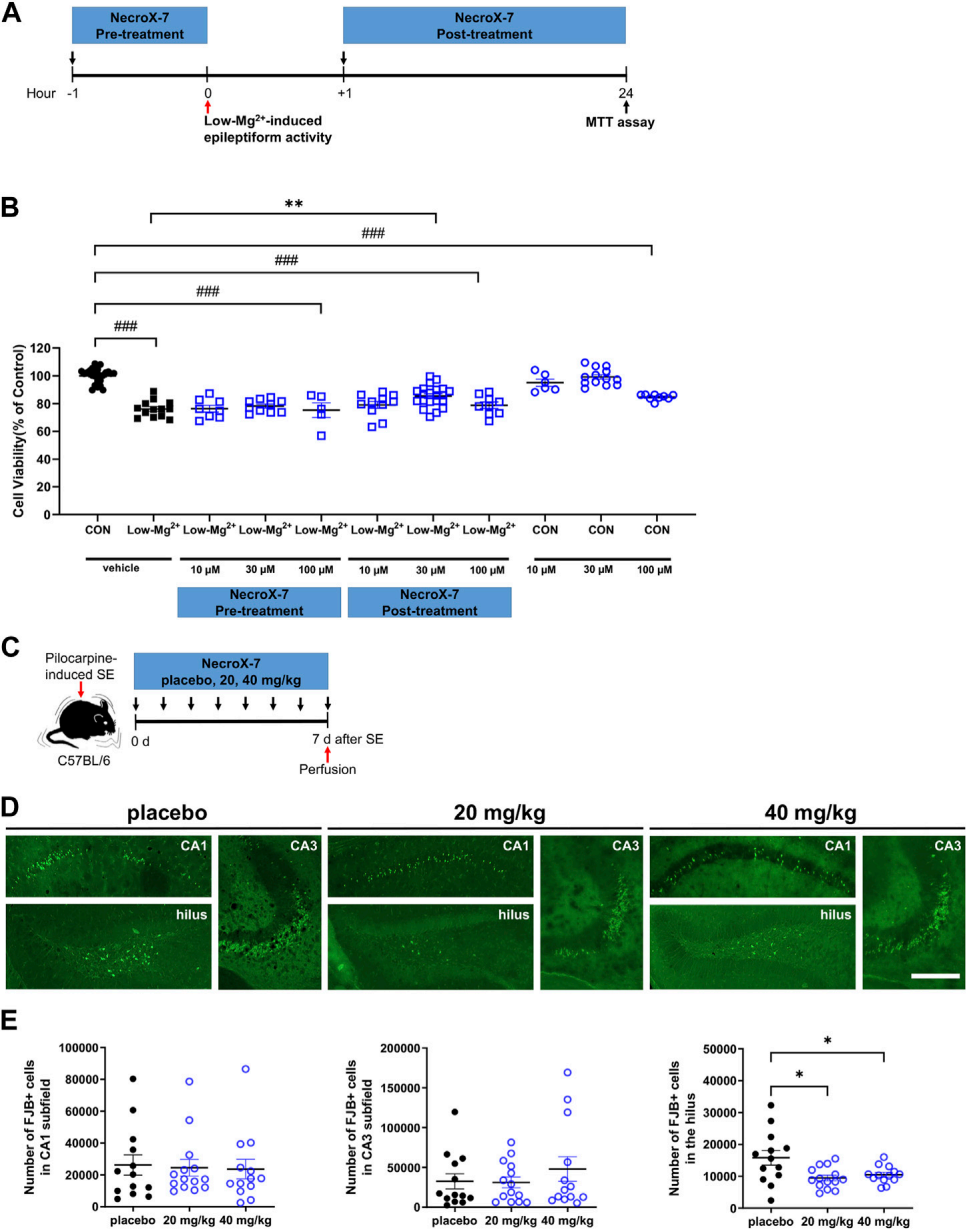
NecroX-7 (30 μM *in vitro*, 20 mg/kg and 40 mg/kg *in vivo*) post-treatment protects cells against 0.1 mM [Mg^2+^]_o_-induced neurotoxicity in cultured rat hippocampal neurons and pilocarpine-induced SE in mouse hippocampal hilus. **(A)** *in vitro* experimental design shown as a timeline. The pretreatment group was treated with NecroX-7 (10 μM, 30 μM, 100 μM) at 1 h before 0.1 mM [Mg^2+^]_o_ treatment for 24 h. The post-treatment group was treated with NecroX-7 (10 μM, 30 μM, 100 μM) at 1 h after 0.1 mM [Mg^2+^]_o_ treatment for 24 h **(B)** A graph showing cell viability in primary cultured hippocampal neurons. One-way ANOVA was performed with Bonferroni post-hoc test, *p* < 0.0001, F_(10, 119)_ = 28.13. Data are shown as mean ± SEM. ^###^ *p* < 0.001 vs. Control, ^***^ *p* < 0.001 vs. low [Mg^2+^]_o_. **(C)** *in vivo* experimental design shown as a timeline. **(D)** Representative microscopic images of each group showing Fluoro-Jade B (FJB)-stained degenerating neurons in hippocampal subfields. Scale bar = 200 μm. **(E)** Graphs showing the number of FJB-positive cells in the hilus, CA1, and CA3 subfields of the hippocampus. Fewer degenerating neurons were observed in the hilus of NecroX-7-treated groups compared with the placebo group. *n* = 13 (placebo), *n* = 14 (20 mg/kg NecroX-7) and *n* = 13 (40 mg/kg NecroX-7). Detailed statistics are as follows. CA1: Kruskal–Wallis test was performed with Dunn’s multiple comparisons test, *p* = 0.98, H = 0.05; CA3: Kruskal–Wallis test was performed with Dunn’s multiple comparisons test, *p* = 0.90, H = 0.21; Hilus: Brown-Forsythe ANOVA was performed with unpaired *t*-test with Welch’s correction test, *p* = 0.02, F_(2, 18.52)_ = 5.28. Data are presented as mean ± SEM. *p < 0.05.

Treatment with the 0.1 mM [Mg^2+^]_o_ in rat primary hippocampal neurons to induce epileptiform discharges showed significant cell death (76.1% ± 1.6%. n = 13, *p* < 0.001) (Figures 1A, B). Pretreatment with NecroX-7 showed no difference in neuronal death caused by 0.1 mM [Mg^2+^]_o_ medium (10 μM of NecroX-7: 76.3% ± 2.4%, n = 8; 30 μM: 78.2% ± 1.4%, n = 10; 100 μM: 75.2% ± 5.3%, n = 5). Post-treatment with NecroX-7 (10 μM and 100 μM) still showed neuronal death (10 μM of NecroX-7: 79.0% ± 2.5%, n = 11; 100 μM: 78.8% ± 2.3%, n = 9). However, NecroX-7 at 30 μM significantly blocked 0.1 mM [Mg^2+^]_o_–induced neurotoxicity (85.4% ± 1.6%, n = 21) (Figure 1B). MK-801 (10 μM), a positive control, showed neuroprotective effects (98.3% ± 1.5%, n = 11) (data not shown). These results indicate that NecroX-7 protects neurons exposed to 0.1 mM [Mg^2+^]_o_ medium when post-treated at 30 μM but not pre-treated.

Before we investigate whether NecroX-7 can attenuate excitotoxic hippocampal damage after pilocarpine-induced SE, we first analyzed behavioral seizure onset and SE onset among placebo, 20 mg/kg, or 40 mg/kg of NecroX-7 post-treatment groups. We found that there were no significant differences regarding the seizure onset (placebo: 18.93 ± 2.19 min vs. 20 mg/kg: 13.60 ± 1.31 min vs. 40 mg/kg: 18.31 ± 1.70 min) and SE onset (placebo: 34.64 ± 3.10 min vs. 20 mg/kg: 26.07 ± 1.85 min vs. 40 mg/kg: 26.38 ± 1.41 min) (Supplementary Figure S1A,C,D), indicating no selection bias in group allocation. Moreover, when we assessed the body weight changes over a 7-day period after pilocarpine injection, we observed a significant recovery in body weight over time without any notable differences among the three groups (Supplementary Figure S1B), demonstrating no drug toxicity. Based on these baseline data, degenerating neurons were then evaluated by FJB staining (Figure 1C). Compared to placebo-treated controls, the number of FJB-positive neurons in the hilus (placebo: 15,807 ± 2,286 vs. 20 mg/kg: 9,478 ± 3,324 vs. 40 mg/kg: 10,508 ± 747) was significantly reduced in both groups of NecroX-7 treatment (Figures 1D, E). However, FJB-positive degenerating neurons were comparable among three groups in the CA1 (placebo: 26,278 ± 6,382 vs. 20 mg/kg: 24,480 ± 5,194 vs. 40 mg/kg: 23,631 ± 6,121) and CA3 (placebo: 32,479 ± 9,507 vs. 20 mg/kg: 31,229 ± 6,779 vs. 40 mg/kg: 48,052 ± 15,483) subfields of the hippocampus (Figures 1D, E), suggesting that the neuroprotective effects of NecroX-7 against pilocarpine-induced SE have region-specific differences. Collectively, our findings demonstrate that NecroX-7 post-treatment after seizure activities can exert hilar neuroprotection.

### Effect of NecroX-7 on low [Mg^2+^]_o_-induced synapse loss in cultured rat hippocampal neurons

**FIGURE 2:**
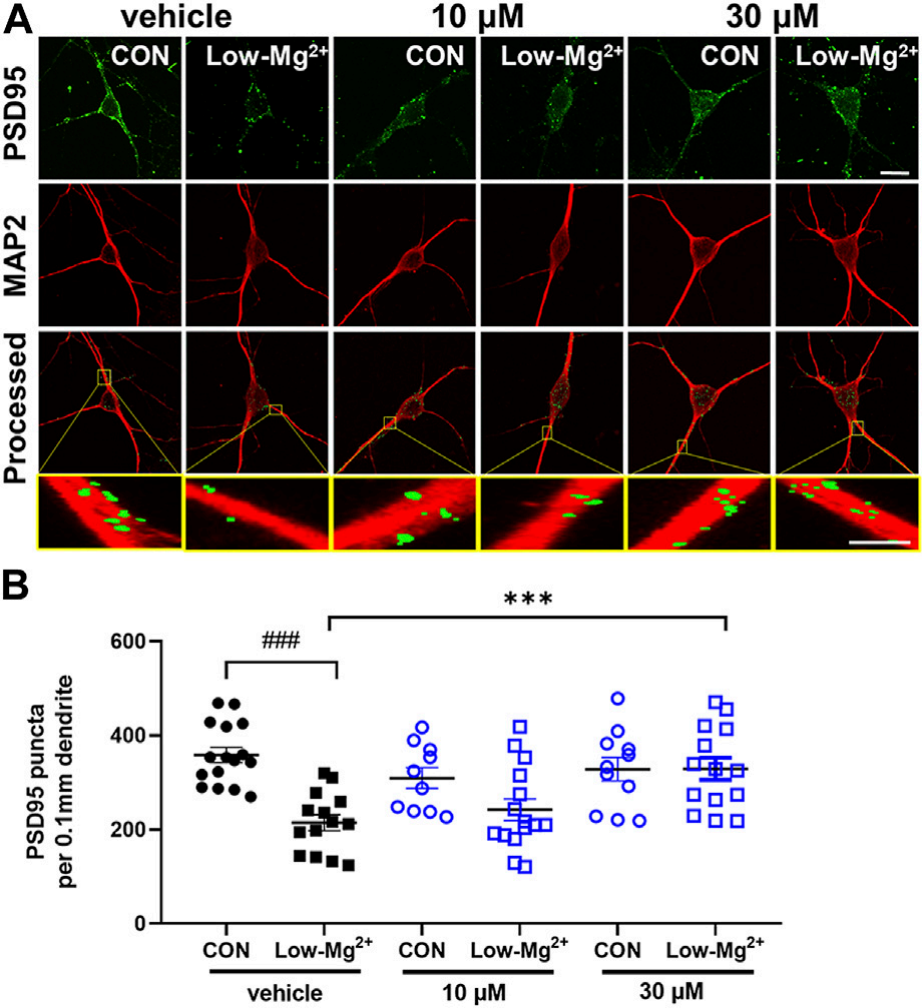
NecroX-7 (30 μM *in vitro*) post-treatment protects against 0.1 mM [Mg^2+^]_o_-induced synaptic loss in cultured rat hippocampal neurons. **(A)** Confocal images show the maximum z-stack of hippocampal neurons expressing MAP2 (red) and PSD95 (green). Scale bar = 10 μm for low magnified images, 2 μm for higher magnified images. **(B)** A graph summarizing the effect of NecroX-7 (10 μM, 30 μM) post-treatment on changes in 0.9 mM [Mg^2+^]_o_ medium or 0.1 mM [Mg^2+^]_o_ medium or changes in the number of PSD95-positive puncta per 0.1 mm dendrite. One-way ANOVA was performed with Bonferroni post-hoc test, *p* < 0.0001, F_(5, 74)_ = 7.63. Data are shown as means ± SEM, ^###^ *p* < 0.001 vs. Control, ^***^ *p* < 0.001 vs. low [Mg^2+^]_o_.

To investigate the effects of NecroX-7 on synapse loss that precedes excitotoxicity, we performed PSD95 immunocytochemistry, which labels excitatory synapses. The number of PSD95 puncta per 0.1 mm dendrite recognized by MAP2 was quantitatively measured (Figure 2A). Treatment with 0.1 mM [Mg^2+^]_o_ medium for 24 h significantly reduced the number of PSD95 puncta by 214.8 ± 17.1 (*n* = 14) (Figure 2B). Pretreatment with MK801 (10 μM) for 30 min significantly prevented synapse loss induced by 0.1 mM [Mg^2+^]_o_ by 335.4 ± 17.9 (*n* = 17), indicating NMDA receptor-dependent synapse loss (data not shown). Post-treatment with NecroX-7 at 10 μM reduced PSD loss by 242.3 ± 22.8 (n = 15) and 30 μM of NecroX-7 significantly prevented PSD loss by 329.3 ± 23.3 (n = 14). This indicates a NecroX-7 concentration-dependent protection against synapse loss elicited by 0.1 mM [Mg^2+^]_o_ (Figure 2B).

### NecroX-7 treatment attenuates seizure-induced neuroinflammation

**FIGURE 3:**
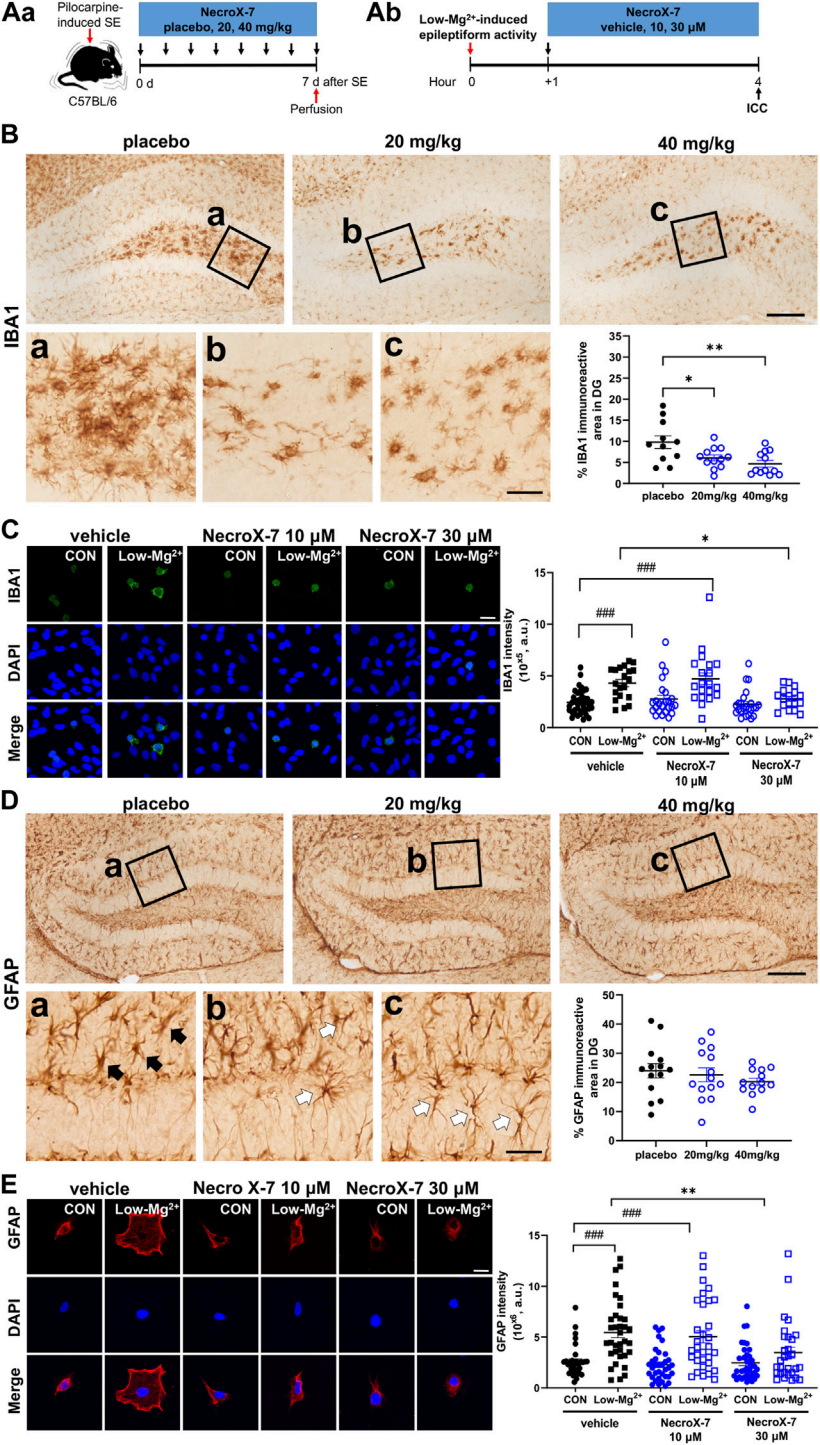
Expression of glial fibrillary acidic protein (GFAP) and ionized calcium-binding adapter molecule 1 (Iba1) by activation of mouse glial cells induced by 0.1 mM [Mg^2+^]_o_ and pilocarpine-induced SE was reduced by NecroX-7 (30 μM *in vitro*, 20 mg/kg and 40 mg/kg *in vivo*) post-treatment. **(Aa)** Experimental design of *in vivo* model shown as a timeline. **(Ab)** Experimental design of *in vitro* model shown as a timeline. **(B)** Representative microscopic images of immunohistochemistry stains of Iba1 in dentate gyrus (DG). Magnified areas in the hilus are indicated by black rectangles **(a-c)** in low magnified images. Note that hypertrophic activated microglia are detected in the hilus in all groups. Scale bar = 200 μm for low magnified images, 50 μm for higher magnified images. A graph showing Iba1-immunoreactive area in DG. Reactive microgliosis was significantly lower in NecroX-7-treated groups (20 mg/kg, 40 mg/kg) compared to the placebo group. *n* = 11 (placebo), *n* = 12 (20 mg/kg) and *n* = 12 (40 mg/kg). One-way ANOVA was performed with Tukey’s multiple comparisons test, *p* = 0.004, F_(2, 32)_ = 6.450. Data are presented as mean ± SEM. *p < 0.05, **p < 0.01. **(C)** Confocal images show the maximum z-stack of hippocampal neurons expressing Iba-1 (green) and DAPI (blue). The graph summarizes the effects of NecroX-7 (10 μM, 30 μM) post-treatment on Iba1 expression caused by 0.9 mM [Mg^2+^]_o_ medium or 0.1 mM [Mg^2+^]_o_ medium. One-way ANOVA was performed with Bonferroni post-hoc test, *p* < 0.0001, F_(5, 139)_ = 9.85. Scale bar = 20 μm. Data are shown as mean ± SEM. ^###^ *p* < 0.001 vs. Control, ^*^ *p* < 0.05, ^**^ *p* < 0.01, ^***^ *p* < 0.001 vs. low [Mg^2+^]_o_. **(D)** Representative microscopic images of immunohistochemistry stains of GFAP in DG. Hypertrophic astrocytes, resembling activated astrocytes, are indicated with black arrows in magnified image **(a)** of the placebo group. Less hypertrophic, ramified, astrocytes are indicated with white arrows in the magnified images **(b, c)** of NecroX-7-treated groups. Scale bar = 200 μm for low magnified images, 50 μm for higher magnified images. A graph showing GFAP-immunoreactive area in DG. Percentages of GFAP-immunoreactive area were comparable between placebo and NecroX-7-treated groups. *n* = 14 (placebo), n = 14 (20 mg/kg) and n = 13 (40 mg/kg). One-way ANOVA was performed with Tukey’s multiple comparisons test, *p* = 0.45, F_(2, 38)_ = 0.81. Data are presented as mean ± SEM. *p < 0.05. **(E)** Confocal images show the maximum z-stack of hippocampal neurons expressing GFAP (red) and DAPI (blue). The graph summarizes the effects of NecroX-7 (10 μM, 30 μM) post-treatment on GFAP expression caused by 0.9 mM [Mg^2+^]_o_ medium or 0.1 mM [Mg^2+^]_o_ medium. One-way ANOVA was performed with Bonferroni post-hoc test, *p* < 0.0001, F_(5, 194)_ = 10.45. Scale bar = 20 μm. Data are shown as mean ± SEM. ^###^ *p* < 0.001 vs. Control, ^*^ *p* < 0.05, ^**^ *p* < 0.01, ^***^ *p* < 0.001 vs. low [Mg^2+^]_o_.

We next examined the effect of NecroX-7 on seizure-induced neuroinflammation by Iba1 and GFAP immunostaining (Figure 3Aa,b). Iba1 immunohistochemistry showed a marked microglial activation in placebo-treated group, whereas 20 mg/kg and 40 mg/kg of NecroX-7 treatment notably downregulated Iba1 immunoreactivities (Figure 3B). Quantitative analysis confirmed that NecroX-7 after SE decreased Iba1-immunoreactive area in DG (placebo: 9.82% ± 1.50% vs. 20 mg/kg: 6.08% ± 0.72% vs. 40 mg/kg: 4.68% ± 0.79%) (Figure 3B). However, quantitative analysis on Iba1-immunoreactive area in CA1 (placebo: 22.93% ± 4.67% vs. 20 mg/kg: 13.55% ± 2.80% vs. 40 mg/kg: 11.95% ± 3.79%) and CA3 (CA3: placebo: 26.31% ± 5.53% vs. 20 mg/kg: 17.63% ± 3.86% vs. 40 mg/kg: 14.64% ± 5.74%) subfields of the hippocampus were not altered by NecroX-7 post-treatment (Supplementary Figure S2), suggesting region-specific differences in NecroX-7-mediated microglial regulation. In addition, rat hippocampal neuron-glia co-cultures also demonstrated that the exposure to 0.1 mM [Mg^2+^]_o_ significantly increased the intensity of Iba1 (4.31 ± 0.35×10^5^, *n* = 20) (Figure 3C). Post-treatment of NecroX-7 at 30 μM significantly reduced the intensity of Iba1 (2.78 ± 0.24×10^5^, n = 17), similar to *in vivo* results (Figure 3C). When we assessed reactive astrocytosis, the GFAP-immunoreactive area in DG (placebo: 24.00% ± 2.43% vs. 20 mg/kg: 22.62% ± 2.35% vs. 40 mg/kg: 20.21% ± 1.23%) was not altered by NecroX-7 post-treatment (Figure 3D). However, hypertrophic GFAP-expressing astrocytes often seen in placebo group (Figure 3D, black arrows) were not frequently detected and instead ramified morphology (Figure 3D, white arrows) was occasionally observed in NecroX-7-treated groups, suggesting morphologic alteration of reactive astrogliosis by NecroX-7 post-treatment. Since we observed morphological changes of reactive astrocytes in the NecroX-7-treated DG, we performed a quantitative analysis using primary cells exposed to low [Mg^2+^]_o_. In hippocampal neuron-glia co-culture, low [Mg^2+^]_o_ markedly increased the size of astrocytes and the intensity of GFAP by 5.45 ± 0.52×10^6^ (n = 35) (Figure 3E). Post-treatment with 30 μM of NecroX-7 dramatically decreased the size of astrocytes and the intensity of GFAP, as shown in Figure 3E (vehicle: 5.45 ± 0.5×10^6^, *n* = 35 vs. 10 μM: 5.03 ± 0.6×10^6^, *n* = 32 vs. 30 μM: 3.47 ± 0.54×10^6^, *n* = 30). These results indicate that NecroX-7 not only decreased GFAP intensity but also significantly restored the morphological changes in astrocytes. Taken all together, our findings demonstrate that NecroX-7 post-treatment can alleviate SE-induced hippocampal neuroinflammation.

### NecroX-7 treatment reduces hippocampal necroptosis marker expression

**FIGURE 4:**
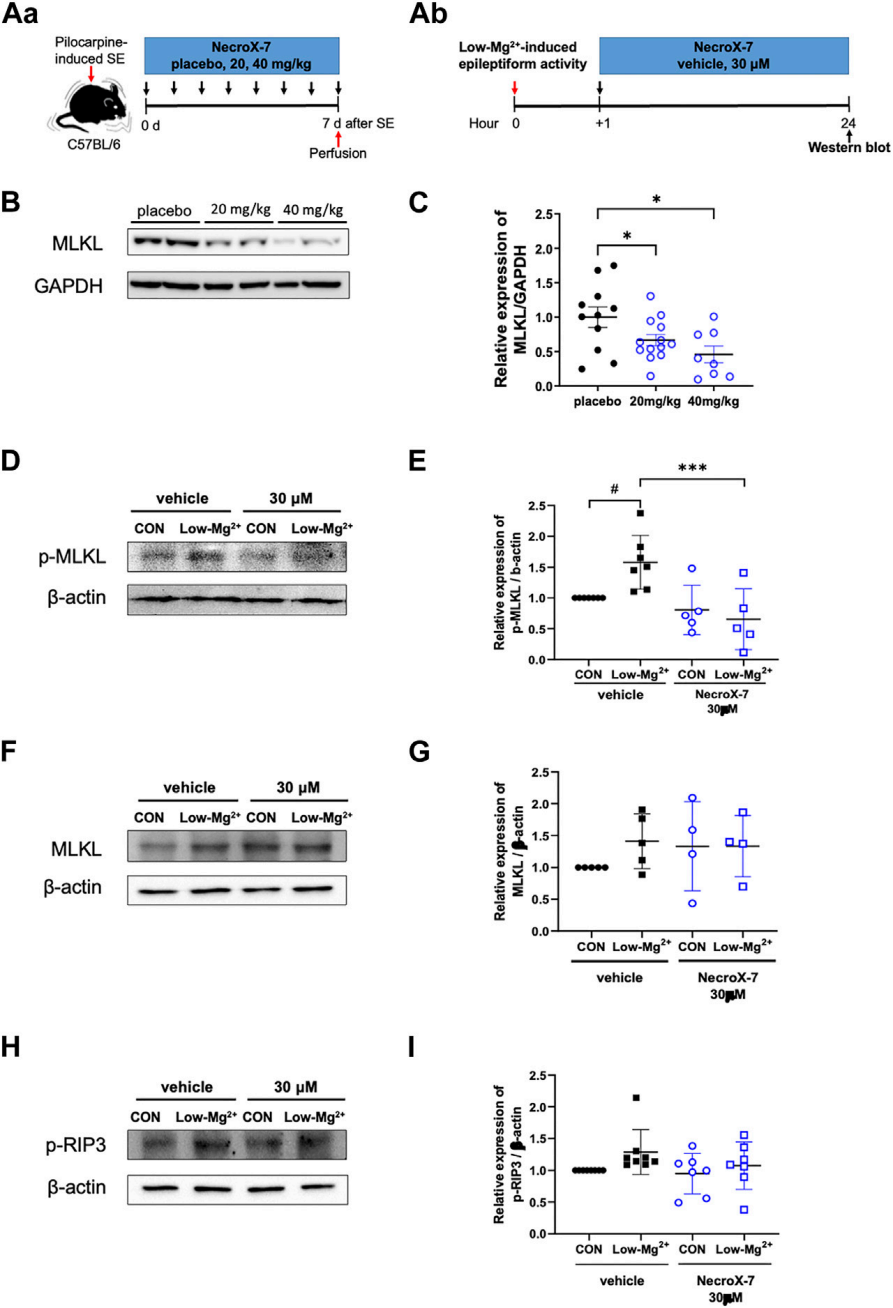
Treatment with NecroX-7 decreases the hippocampal necroptosis marker expression. **(A)** Experimental design shown as a timeline, *in vivo* **(Aa)** and *in vitro* **(Ab)**. **(B)** Representative western blot image of MLKL expression in the hippocampus. GAPDH expression was used as a loading control. **(C)** A graph comparing relative expression of MLKL/GAPDH. MLKL expression was significantly lower in the NecroX-7-treated groups compared to the placebo group. n = 11 (placebo), n = 13 (20 mg/kg) and n = 8 (40 mg/kg). One-way ANOVA analysis with Newman-Keuls multiple comparisons test, *p* = 0.02, F_(2, 29)_ = 4.83. Data are presented as mean ± SEM. *p < 0.05. **(D, F, H)** Representative western blot images of phosphorylated MLKL (p-MLKL), MLKL, and phosphorylated RIP3 (p-RIP3) expression in cultured rat hippocampal neurons. β-actin expression was used as a loading control**. (E, G, I)** Graphs comparing relative expression of p-MLKL/β-actin **(E)**, MLKL/β-actin **(G),** and p-RIP3/β-actin **(I)**. p-MLKL expression showed a significant increase after 0.1 mM [Mg^2+^]_o_-induced epileptiform activity. Notably, the increase in p-MLKL by 0.1 mM [Mg^2+^]_o_-induced epileptiform activity was significantly reduced by NecroX-7 post-treatment at 30 μM. *n* = 7 (vehicle CON), *n* = 7 (vehicle Low-Mg^2+^), *n* = 5 (30 μM CON), and *n* = 5 (30 μM Low-Mg^2+^) for p-MLKL expression. One-way ANOVA analysis with Bonferroni multiple comparisons test, *p* < 0.0001, F_(3, 20)_ = 7.38. Data are presented as mean ± SEM. ^#^ *p* < 0.05 vs. Control, ^***^ *p* < 0.001 vs. low [Mg^2+^]_o_. For MLKL, *n* = 5 (vehicle CON), *n* = 5 (vehicle Low-Mg^2+^), *n* = 4 (30 μM CON) and *n* = 4 (30 μM Low-Mg^2+^). For p-RIP3, *n* = 8 (vehicle CON), *n* = 8 (vehicle Low-Mg^2+^), *n* = 7 (30 μM CON) and *n* = 7 (30 μM Low-Mg^2+^).

Since NecroX-7 alleviated neuroinflammation and neuronal death, we tried to evaluate the nature of NecroX-7-mediated neuroprotection after acute seizures *in vivo* (Figure 4Aa) and *in vitro* (Figure 4Ab). On the 7^th^ day after SE, our western blot analysis demonstrated that the hippocampal MLKL expression (placebo: 1.00 ± 0.15 vs. 20 mg/kg: 0.67 ± 0.08 vs. 40 mg/kg: 0.46 ± 0.12), a marker for necroptosis, was significantly decreased in groups of both 20 mg/kg and 40 mg/kg of NecroX-7 post-treatment, compared to placebo group (Figures 4B, C). In addition, post-treatment of hippocampal neurons exposed to 0.1 mM [Mg^2+^]_o_ showed a significant induction of phopho-MLKL (p-MLKL) expression, which was markedly decreased by the treatment of 30 μM of NecroX-7 (Figures 4D, E). The expression of MLKL (Figures 4F, G) and phospho-RIP3 (Figures 4H, I) also demonstrated an increasing trend in the cells exposed to 0.1 mM [Mg^2+^]_o_. Collectively, our results suggest that NecroX-7 treatment after SE can contribute to the reduction of seizure-induced hippocampal necroptosis.

## Discussion

Despite longstanding rigorous efforts to develop effective anti-epileptic drugs, epilepsy remains intractable in one-third of its patients, thereby still requiring novel drugs. In this study, we investigated a small molecule, NecroX-7, as a potential therapeutic agent for TLE. Administration of NecroX-7 after acute seizures demonstrated neuroprotective and anti-inflammatory effects on hippocampal neurons in both *in vivo* and *in vitro* models of TLE. Additionally, we demonstrated that NecroX-7 could reduce seizure-induced necroptosis marker expressions in the hippocampus.

In the present study, we proposed that necroptosis could play a role in seizure-induced cell deaths, which was prevented by NecroX-7 treatment. Necroptosis, a type of cell death also known as programmed necrosis, can be induced when TNFα binds to its receptor, TNFR1, and activates RIP1/3 to phosphorylate MLKL, an important marker of necroptosis (Yu et al., 2021). Phosphorylated MLKL then forms an oligomer and binds to the cell membrane, causing Ca^2+^ and Na^+^ influx, and increasing extracellular permeation of cellular components including DAMPs, which eventually leads to cell death (Du et al., 2022). Since SE is an inflammatory condition and cell deaths in SE in part showed necrotic morphologies (Fujikawa et al., 2000a; Dingledine et al., 2014; Wang et al., 2017b; Cai et al., 2017; Fricker et al., 2018; Du et al., 2022), involvement of necroptosis has been assumed. Indeed, we reported the first evidence that acute seizures can cause MLKL-mediated necroptosis in the hippocampus, in addition to the involvement of truncated neogenin in this process (Choi et al., 2021). We further sought to find an anti-necroptotic agent and found a novel small molecule, NecroX-7, that could decrease MLKL and p-MLKL expression after acute seizures. In other studies, inhibition of MLKL reduced SE-related brain damage and improved cognitive performance (Wang et al., 2017a; Jia et al., 2019), supporting our data. Taken all together, we provide a new lead compound, NecroX-7, as a potential anti-epileptic drug by demonstrating the alleviation of excitotoxic necroptosis.

We also demonstrated that NecroX-7 could effectively regulate seizure-induced neuroinflammation. We found that NecroX-7 treatment significantly reduced the low [Mg^2+^]o-induced activation of microglia and astrocytes, together with the restoration of hypertrophic astrocytic morphology. Our *in vivo* and *in vitro* results suggest an anti-inflammatory role of NecroX-7 in TLE. In diverse experimental models of degenerative and inflammatory diseases including GVHD, chemotherapy-associated mucositis, atherosclerosis, and myocardial, renal, and hepatic ischemia-reperfusion injuries, NecroX-7 decreased the expression of pro-inflammatory cytokines or damage-associated molecular pattern (DAMPs), i.e., TNFα, IL-1R, IL-6, iNOS, MCP-1, or HMGB1 (Im et al., 2015; Grootaert et al., 2016; Jin et al., 2016; Lee et al., 2016; Hwang et al., 2018; Im et al., 2019), in line with our findings of NecroX-7-mediated anti-inflammation. Moreover, NecroX-7 controlled inflammatory cellular pathways such as NF-κB, JNK 1/2, and p38 signaling (Park et al., 2013; Chung et al., 2015; Grootaert et al., 2016; Hwang et al., 2018; Kim et al., 2021). As the inhibition of cytoplasmic and extracellular redistribution of HMGB1 from the nucleus is considered one of the major modes of action of NecroX-7 (Park et al., 2012; Im et al., 2015; Grootaert et al., 2016; Jin et al., 2016; Lee et al., 2016; Hwang et al., 2018; Im et al., 2019; Kim et al., 2021), it is plausible that in SE, blocking extracellular leakage of DAMPs by NecroX-7 may interfere with the initiation of inflammatory pathways and the production of pro-inflammatory cytokines including TNFα, attenuating necroptosis and thereby promoting hippocampal neuroprotection.

NecroX-7 has an advantage when it comes to drug development because it is bioavailable with oral administration and is non-toxic. Studies of IV administration of single and multiple doses of NecroX-7 to a healthy male population showed no significant side effects and it was confirmed to have optimal pharmacokinetic properties, such as dose proportionality, fast tissue distribution, and long t_½_, for a once-daily regimen (Kim et al., 2017; Kim et al., 2020). Since first-in-human trials have been conducted, a future direction will be to test the efficacy of NecroX-7 in TLE patients. A more elaborate study design will be required to determine the appropriate dosage of NecroX-7 for TLE as wide dose ranges of 0.3–30 mg/kg (i.v.), and 20-50 mg/kg (p.o.) in mice have been reported to show therapeutic efficacy in various diseases (Park et al., 2013; Chung et al., 2015; Im et al., 2019). We also reported that 20-40 mg/kg of NecroX-7 had anti-necroptotic and anti-inflammatory effects in a mouse model of TLE. Given that the blood-brain barrier (BBB) hinders efficient drug delivery to the cells in the brain, modification of the drug structure to enhance BBB penetration or high doses of NecroX-7 may be necessary to demonstrate therapeutic efficacy in the context of epilepsy. In addition, testing a variety of different cell types will be also required to provide comprehensive information about the efficacy and potential side effects of NecroX-7, especially when high doses of NecroX-7 need to be treated. However, the easy administration and good tolerability of NecroX-7 make it a more promising candidate as a novel anti-epileptic drug.

For future clinical trials of NecroX-7 in epilepsy, it will be valuable to compare the therapeutic efficacies of multiple drugs that can regulate various aspects of necroptosis, i.e., necrostatin-1 (Nec-1), dabrafenib, or necrosulfonamide (NSA), with respect to that of NecroX-7. Necrostatin-1 (Nec-1) is an ATP-competitive small molecule and allosterically inhibits RIP1, an upstream kinase of MLKL (Degterev et al., 2008). Dabrafenib, an FDA-approved immunotherapy agent for melanomas, is originally a B-RAF^V600E^ inhibitor, but was later found to selectively inhibit RIP3 (Sugaya et al., 2019). Finally, NSA, another small molecule, is known to block MLKL oligomerization and activation (Yan et al., 2017; Bansal et al., 2019). As our results suggest that NecroX-7 can affect the steps associated with MLKL in the hippocampus, it will be interesting to evaluate differential impacts of targeting each key signaling molecule of necroptosis on neuroprotection after acute seizures.

In our study, while NecroX-7 successfully protected neuronal death in the hilus, pyramidal neurons in the CA1 and CA3 subfields could not be saved after pilocarpine-induced SE. This regional difference in cell death might be associated with region-specific alterations in microglial activation as we found that Iba1 immunoreactivity was alleviated only in the hilus. Alternatively, it may be related with different biochemical characteristics of each cell type, leading to altered response to environmental stimuli and NecroX-7 treatment. For instance, hilar interneurons express C-C motif chemokine receptor 8 (CCR8), which can inhibit dexamethasone-induced apoptosis (Spinetti et al., 2003), while pyramidal neurons do not (Liu et al., 2007). Despite speculative at the moment, NecroX-7 might boost the protective effect of CCR8, thus salvaging hilar interneurons. This hypothesis warrants further comprehensive investigation in the future as transcriptomic analysis between hilar interneurons and pyramidal neurons for the identification of differentially expressed candidates after acute seizures and then after NecroX-7 administration, respectively.

In summary, we propose NecroX-7 as a potential therapeutic agent for TLE by demonstrating the neuroprotective and anti-inflammatory effects *in vitro* and *in vivo*. In addition, we suggest that NecroX-7 after acute seizures can mediate anti-necroptosis in the hippocampus.

## Data Availability

The original contributions presented in the study are included in the article/Supplementary Material, further inquiries can be directed to the corresponding authors.

## References

[B1] Bansal N. Sciabola S. Bhisetti G. (2019). Understanding allosteric interactions in hMLKL protein that modulate necroptosis and its inhibition. Sci. Rep. 9, 16853. 10.1038/s41598-019-53078-5 31727943 PMC6856060

[B2] Brulet R. Zhu J. Aktar M. Hsieh J. Cho K. O. (2017). Mice with conditional NeuroD1 knockout display reduced aberrant hippocampal neurogenesis but no change in epileptic seizures. Exp. Neurol. 293, 190–198. 10.1016/j.expneurol.2017.04.005 28427858 PMC5503142

[B3] Cai Q. Gan J. Luo R. Qu Y. Li S. Wan C. (2017). The role of necroptosis in status epilepticus-induced brain injury in juvenile rats. Epilepsy Behav. 75, 134–142. 10.1016/j.yebeh.2017.05.025 28863321

[B4] Cho K. O. Jeong K. H. Cha J. H. Kim S. Y. (2020). Spatiotemporal expression of RCAN1 and its isoform RCAN1-4 in the mouse hippocampus after pilocarpine-induced status epilepticus. Korean J. Physiol. Pharmacol. 24, 81–88. 10.4196/kjpp.2020.24.1.81 31908577 PMC6940495

[B5] Cho K. O. Kim J. Y. Jeong K. H. Lee M. Y. Kim S. Y. (2019). Increased expression of vascular endothelial growth factor-C and vascular endothelial growth factor receptor-3 after pilocarpine-induced status epilepticus in mice. Korean J. Physiol. Pharmacol. 23, 281–289. 10.4196/kjpp.2019.23.4.281 31297012 PMC6609264

[B6] Cho K. O. Lybrand Z. R. Ito N. Brulet R. Tafacory F. Zhang L. (2015). Aberrant hippocampal neurogenesis contributes to epilepsy and associated cognitive decline. Nat. Commun. 6, 6606. 10.1038/ncomms7606 25808087 PMC4375780

[B7] Choi I.-Y. Cho M.-L. Cho K.-O. (2022). Interleukin-17A mediates hippocampal damage and aberrant neurogenesis contributing to epilepsy-associated anxiety. Front. Mol. Neurosci. 15, 917598. 10.3389/fnmol.2022.917598 35875667 PMC9298510

[B8] Choi I. Y. Shim J. H. Kim M. H. Yu W. D. Kim Y. J. Choi G. (2021). Truncated neogenin promotes hippocampal neuronal death after acute seizure. Neuroscience 470, 78–87. 10.1016/j.neuroscience.2021.06.039 34245840

[B9] Choi J. M. Park K. M. Kim S. H. Hwang D. W. Chon S. H. Lee J. H. (2010). Effect of necrosis modulator necrox-7 on hepatic ischemia-reperfusion injury in beagle dogs. Transpl. Proc. 42, 3414–3421. 10.1016/j.transproceed.2010.08.050 21094788

[B10] Chung H. K. Kim Y. K. Park J. H. Ryu M. J. Chang J. Y. Hwang J. H. (2015). The indole derivative NecroX-7 improves nonalcoholic steatohepatitis in ob/ob mice through suppression of mitochondrial ROS/RNS and inflammation. Liver Int. 35, 1341–1353. 10.1111/liv.12741 25443620

[B11] Degterev A. Hitomi J. Germscheid M. Chen I. L. Korkina O. Teng X. (2008). Identification of RIP1 kinase as a specific cellular target of necrostatins. Nat. Chem. Biol. 4, 313–321. 10.1038/nchembio.83 18408713 PMC5434866

[B12] Dingledine R. Varvel N. H. Dudek F. E. (2014). When and how do seizures kill neurons, and is cell death relevant to epileptogenesis? Adv. Exp. Med. Biol. 813, 109–122. 10.1007/978-94-017-8914-1_9 25012371 PMC4624106

[B13] Du K. He M. Zhao D. Wang Y. Ma C. Liang H. (2022). Mechanism of cell death pathways in status epilepticus and related therapeutic agents. Biomed. Pharmacother. 149, 112875. 10.1016/j.biopha.2022.112875 35367755

[B14] Fricker M. Tolkovsky A. M. Borutaite V. Coleman M. Brown G. C. (2018). Neuronal cell death. Physiol. Rev. 98, 813–880. 10.1152/physrev.00011.2017 29488822 PMC5966715

[B15] Fujikawa D. G. Shinmei S. S. Cai B. (2000a). Kainic acid-induced seizures produce necrotic, not apoptotic, neurons with internucleosomal DNA cleavage: Implications for programmed cell death mechanisms. Neuroscience 98, 41–53. 10.1016/s0306-4522(00)00085-3 10858610

[B16] Fujikawa D. G. Shinmei S. S. Cai B. (2000b). Seizure-induced neuronal necrosis: Implications for programmed cell death mechanisms. Epilepsia 41 (Suppl. 6), S9–S13. 10.1111/j.1528-1157.2000.tb01549.x 10999512

[B17] Grootaert M. O. J. Schrijvers D. M. Van Spaendonk H. Breynaert A. Hermans N. Van Hoof V. O. (2016). NecroX-7 reduces necrotic core formation in atherosclerotic plaques of Apoe knockout mice. Atherosclerosis 252, 166–174. 10.1016/j.atherosclerosis.2016.06.045 27425215

[B18] Hong G. P. Kim M. H. Kim H. J. (2021). Sex-related differences in glial fibrillary acidic protein-positive GABA regulate neuropathology following pilocarpine-induced status epilepticus. Neuroscience 472, 157–166. 10.1016/j.neuroscience.2021.08.002 34400247

[B19] Hong N. Park J. S. Kim H. J. (2022). Synapto-protective effect of lithium on HIV-1 Tat-induced synapse loss in rat hippocampal cultures. Anim. Cells Syst. Seoul. 26, 1–9. 10.1080/19768354.2021.2018044 35308128 PMC8928815

[B20] Hwang I. C. Kim J. Y. Kim J. H. Lee J. E. Seo J. Y. Lee J. W. (2018). Therapeutic potential of a novel necrosis inhibitor, 7-amino-indole, in myocardial ischemia-reperfusion injury. Hypertension 71, 1143–1155. 10.1161/HYPERTENSIONAHA.117.09405 29661840 PMC5959205

[B21] Im K. I. Kim N. Lim J. Y. Nam Y. S. Lee E. S. Kim E. J. (2015). The free radical scavenger NecroX-7 attenuates acute graft-versus-host disease via reciprocal regulation of Th1/regulatory T cells and inhibition of HMGB1 release. J. Immunol. 194, 5223–5232. 10.4049/jimmunol.1402609 25911749 PMC4432727

[B22] Im K. I. Nam Y. S. Kim N. Song Y. Lee E. S. Lim J. Y. (2019). Regulation of HMGB1 release protects chemoradiotherapy-associated mucositis. Mucosal Immunol. 12, 1070–1081. 10.1038/s41385-019-0132-x 30647411

[B23] Jang H. J. Kim J. E. Jeong K. H. Lim S. C. Kim S. Y. Cho K. O. (2019). The neuroprotective effect of hericium erinaceus extracts in mouse Hippocampus after pilocarpine-induced status epilepticus. Int. J. Mol. Sci. 20, 859. 10.3390/ijms20040859 30781501 PMC6413080

[B24] Jeong K. H. Lee K. E. Kim S. Y. Cho K. O. (2011). Upregulation of Kruppel-like factor 6 in the mouse hippocampus after pilocarpine-induced status epilepticus. Neuroscience 186, 170–178. 10.1016/j.neuroscience.2011.02.046 21362463

[B25] Jia R. Jia N. Yang F. Liu Z. Li R. Jiang Y. (2019). Hydrogen alleviates necroptosis and cognitive deficits in lithium-pilocarpine model of status epilepticus. Cell Mol. Neurobiol. 39, 857–869. 10.1007/s10571-019-00685-5 31089833 PMC11462845

[B26] Jin S. A. Kim S. K. Seo H. J. Jeong J. Y. Ahn K. T. Kim J. H. (2016). Beneficial effects of necrosis modulator, indole derivative NecroX-7, on renal ischemia-reperfusion injury in rats. Transpl. Proc. 48, 199–204. 10.1016/j.transproceed.2015.12.018 26915868

[B27] Kim E. Hwang I. Lee S. Oh J. Chung H. Jin M. (2020). Pharmacokinetics and tolerability of LC28-0126, a novel necrosis inhibitor, after multiple ascending doses: A phase I randomized, double-blind, placebo-controlled study in healthy male subjects. Clin. Ther. 42, 1946–1954.e2. 10.1016/j.clinthera.2020.08.011 32980184

[B28] Kim G. Lee H. S. Oh B. J. Kwon Y. Kim H. Ha S. (2021). Protective effect of a novel clinical-grade small molecule necrosis inhibitor against oxidative stress and inflammation during islet transplantation. Am. J. Transpl. 21, 1440–1452. 10.1111/ajt.16323 32978875

[B29] Kim H. J. Koo S. Y. Ahn B. H. Park O. Park D. H. Seo D. O. (2010). NecroX as a novel class of mitochondrial reactive oxygen species and ONOO(-) scavenger. Arch. Pharm. Res. 33, 1813–1823. 10.1007/s12272-010-1114-4 21116785

[B30] Kim H. J. Waataja J. J. Thayer S. A. (2008). Cannabinoids inhibit network-driven synapse loss between hippocampal neurons in culture. J. Pharmacol. Exp. Ther. 325, 850–858. 10.1124/jpet.107.131607 18310474 PMC2398764

[B31] Kim H. J. Yang J. S. Yoon S. H. Sim S. J. Lee J. (2016). Reversible and multi-cyclic protein-protein interaction in bacterial cellulosome-mimic system using rod-shaped viral nanostructure. Korean J. Physiol. Pharmacol. 20, 101–106. 10.1016/j.jbiotec.2016.01.033 26820321

[B32] Kim J. E. Cho K. O. (2018). The pilocarpine model of temporal lobe epilepsy and EEG monitoring using radiotelemetry system in mice. J. Vis. Exp., 56831. 10.3791/56831 29553531 PMC5931368

[B33] Kim S. Chung H. Lee S. Cho S. H. Cho H. J. Kim S. H. (2017). Pharmacokinetics and safety of a single dose of the novel necrosis inhibitor LC28-0126 in healthy male subjects. Br. J. Clin. Pharmacol. 83, 1205–1215. 10.1111/bcp.13213 28002882 PMC5427235

[B34] Lee J. H. Park K. M. Lee Y. J. Kim J. H. Kim S. H. (2016). A new chemical compound, NecroX-7, acts as a necrosis modulator by inhibiting high-mobility group box 1 protein release during massive ischemia-reperfusion injury. Transpl. Proc. 48, 3406–3414. 10.1016/j.transproceed.2016.09.046 27931589

[B35] Liu J. X. Cao X. Tang Y. C. Liu Y. Tang F. R. (2007). CCR7, CCR8, CCR9 and CCR10 in the mouse hippocampal CA1 area and the dentate gyrus during and after pilocarpine-induced status epilepticus. J. Neurochem. 100, 1072–1088. 10.1111/j.1471-4159.2006.04272.x 17181556

[B36] Park J. H. Seo K. S. Tadi S. Ahn B. H. Lee J. U. Heo J. Y. (2013). An indole derivative protects against acetaminophen-induced liver injury by directly binding to N-acetyl-p-benzoquinone imine in mice. Antioxid. Redox Signal 18, 1713–1722. 10.1089/ars.2012.4677 23121402 PMC3619205

[B37] Park J. Park E. Ahn B. H. Kim H. J. Park J. H. Koo S. Y. (2012). NecroX-7 prevents oxidative stress-induced cardiomyopathy by inhibition of NADPH oxidase activity in rats. Toxicol. Appl. Pharmacol. 263, 1–6. 10.1016/j.taap.2012.05.014 22659508

[B38] Racine R. J. (1972). Modification of seizure activity by electrical stimulation. II. Motor seizure. Electroencephalogr. Clin. Neurophysiol. 32, 281–294. 10.1016/0013-4694(72)90177-0 4110397

[B39] Sankar R. Shin D. H. Liu H. Mazarati A. Pereira De Vasconcelos A. Wasterlain C. G. (1998). Patterns of status epilepticus-induced neuronal injury during development and long-term consequences. J. Neurosci. 18, 8382–8393. 10.1523/JNEUROSCI.18-20-08382.1998 9763481 PMC6792849

[B40] Sloviter R. S. Dean E. Sollas A. L. Goodman J. H. (1996). Apoptosis and necrosis induced in different hippocampal neuron populations by repetitive perforant path stimulation in the rat. J. Comp. Neurol. 366, 516–533. 10.1002/(SICI)1096-9861(19960311)366:3<516::AID-CNE10>3.0.CO;2-N 8907362

[B41] Spinetti G. Bernardini G. Camarda G. Mangoni A. Santoni A. Capogrossi M. C. (2003). The chemokine receptor CCR8 mediates rescue from dexamethasone-induced apoptosis via an ERK-dependent pathway. J. Leukoc. Biol. 73, 201–207. 10.1189/jlb.0302105 12525579

[B42] Steve T. A. Jirsch J. D. Gross D. W. (2014). Quantification of subfield pathology in hippocampal sclerosis: A systematic review and meta-analysis. Epilepsy Res. 108, 1279–1285. 10.1016/j.eplepsyres.2014.07.003 25107686

[B43] Sugaya T. Kanno H. Matsuda M. Handa K. Tateda S. Murakami T. (2019). B-RAF(V600E) inhibitor dabrafenib attenuates RIPK3-mediated necroptosis and promotes functional recovery after spinal cord injury. Cells 8, 1582. 10.3390/cells8121582 31817643 PMC6953123

[B44] Tai X. Y. Bernhardt B. Thom M. Thompson P. Baxendale S. Koepp M. (2018). Review: Neurodegenerative processes in temporal lobe epilepsy with hippocampal sclerosis: Clinical, pathological and neuroimaging evidence. Neuropathol. Appl. Neurobiol. 44, 70–90. 10.1111/nan.12458 29288503

[B45] Tellez-Zenteno J. F. Hernandez-Ronquillo L. (2012). A review of the epidemiology of temporal lobe epilepsy. Epilepsy Res. Treat. 2012, 630853. 10.1155/2012/630853 22957234 PMC3420432

[B46] Vezzani A. French J. Bartfai T. Baram T. Z. (2011). The role of inflammation in epilepsy. Nat. Rev. Neurol. 7, 31–40. 10.1038/nrneurol.2010.178 21135885 PMC3378051

[B47] Wang J. Li Y. Huang W. H. Zeng X. C. Li X. H. Li J. (2017a). The protective effect of aucubin from eucommia ulmoides against status epilepticus by inducing autophagy and inhibiting necroptosis. Am. J. Chin. Med. 45, 557–573. 10.1142/S0192415X17500331 28387136

[B48] Wang J. Liu Y. Li X. H. Zeng X. C. Li J. Zhou J. (2017b). Curcumin protects neuronal cells against status-epilepticus-induced hippocampal damage through induction of autophagy and inhibition of necroptosis. Can. J. Physiol. Pharmacol. 95, 501–509. 10.1139/cjpp-2016-0154 28177687

[B49] World Health Organization (2022). Epilepsy fact sheet [online]. Available at: https://www.who.int/news-room/fact-sheets/detail/epilepsy (Accessed July 9, 2022).

[B50] Yan B. Liu L. Huang S. Ren Y. Wang H. Yao Z. (2017). Discovery of a new class of highly potent necroptosis inhibitors targeting the mixed lineage kinase domain-like protein. Chem. Commun. (Camb) 53, 3637–3640. 10.1039/c7cc00667e 28267172

[B51] Yu Z. Jiang N. Su W. Zhuo Y. (2021). Necroptosis: A novel pathway in neuroinflammation. Front. Pharmacol. 12, 701564. 10.3389/fphar.2021.701564 34322024 PMC8311004

