# Supplementary figures and images for: Alleviation of hippocampal necroptosis and neuroinflammation by NecroX-7 treatment after acute seizures
Source: Front Pharmacol. 2023 Aug 2;14:1187819. doi: 10.3389/fphar.2023.1187819 (PMC10433749; doi:10.3389/fphar.2023.1187819)

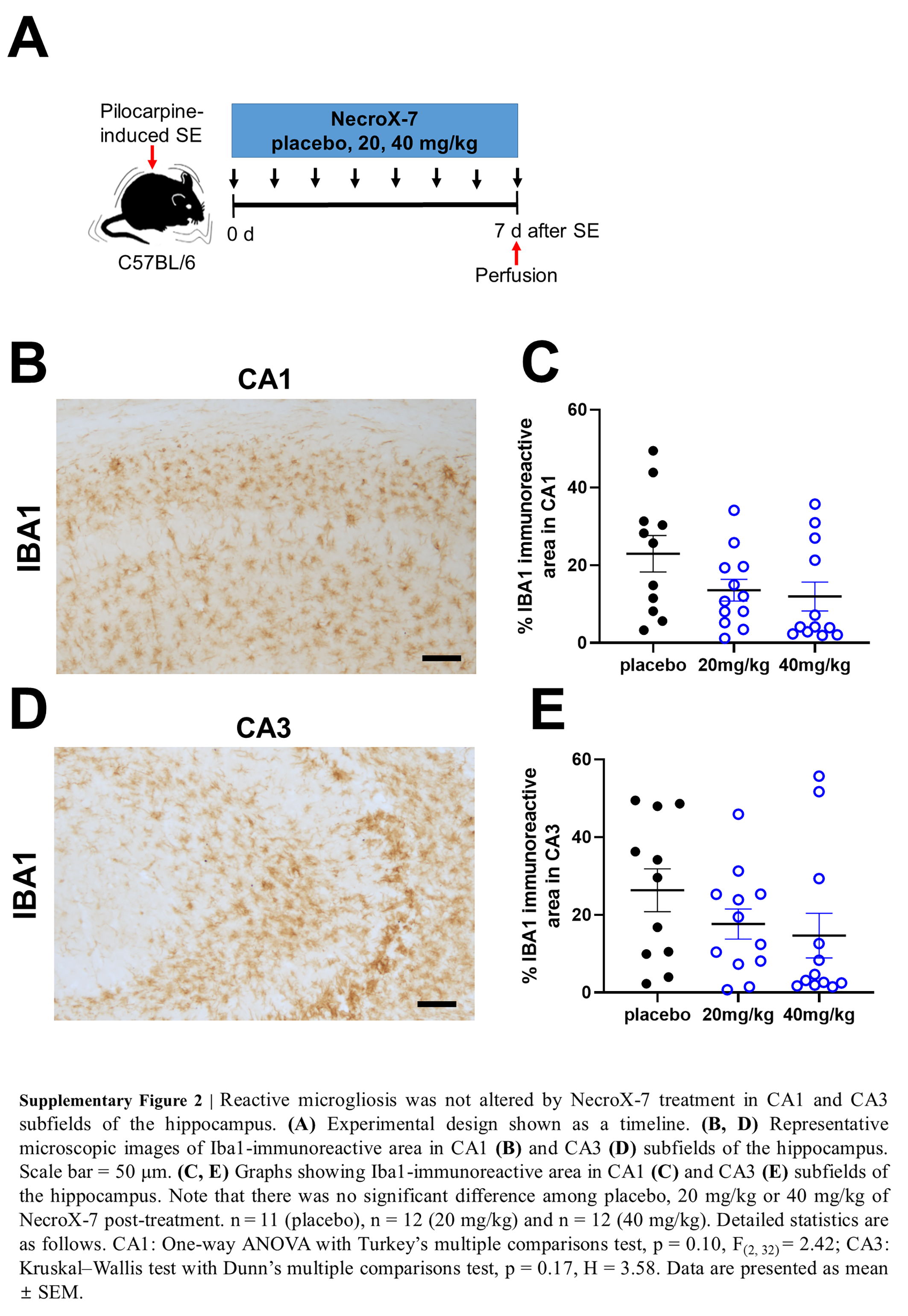

Supplement: Supplementary file 1 [file Image2.TIF]

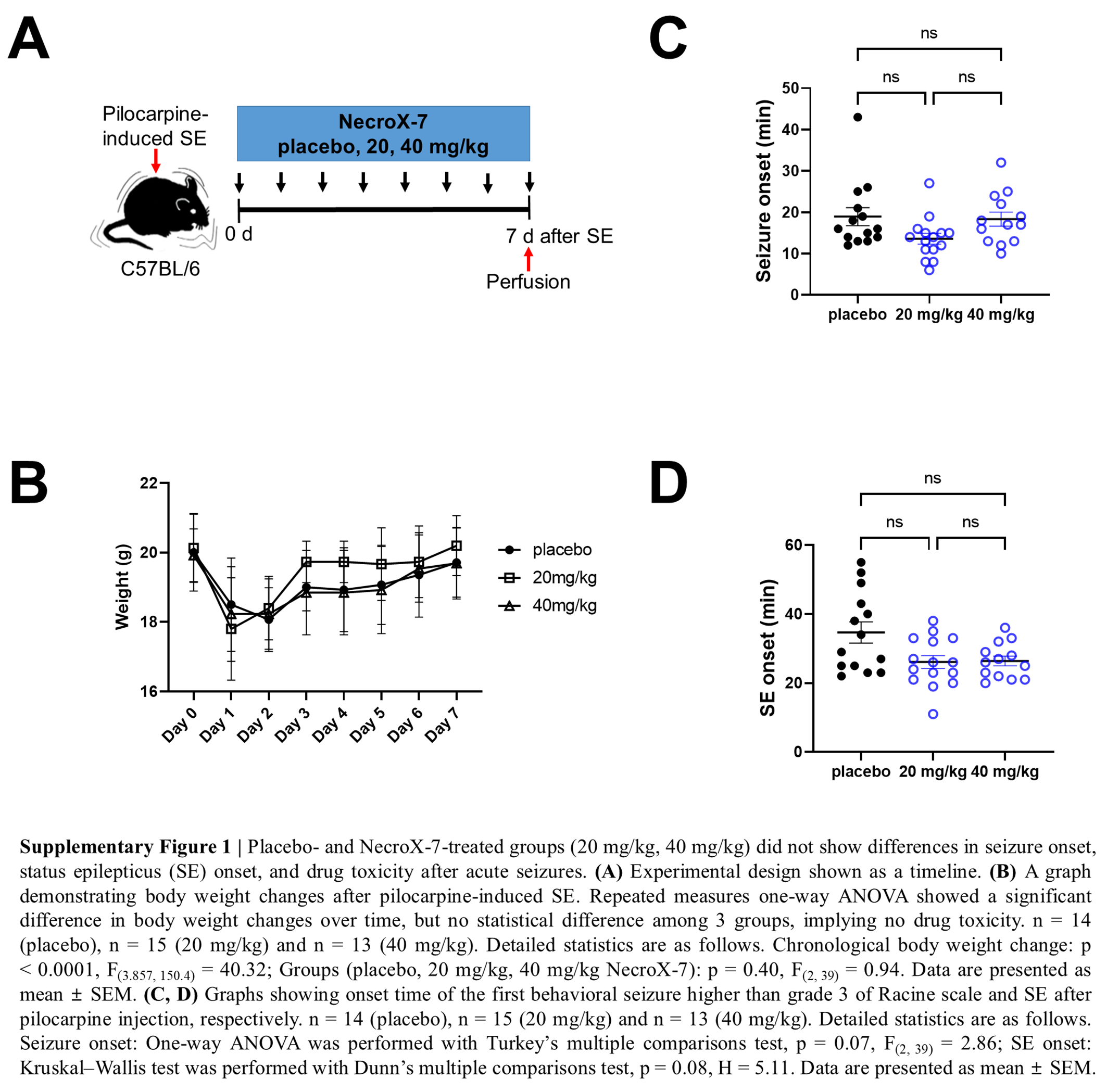

Supplement: Supplementary file 2 [file Image1.TIF]
